# Examining the Association Between Equity-Related Factors and EQ-5D-3L Health Utilities of Patients with Cancer

**DOI:** 10.3390/curroncol32110645

**Published:** 2025-11-19

**Authors:** Teresa C. O. Tsui, Rebecca E. Mercer, Eleanor M. Pullenayegum, Kelvin K. W. Chan

**Affiliations:** 1 Sunnybrook Research Institute, Toronto, ON M4N 3M5, Canada; teresa.tsui@alumni.utoronto.ca (T.C.O.T.); rebecca.mercer@sunnybrook.ca (R.E.M.); 2 Dalla Lana School of Public Health, University of Toronto, Toronto, ON M5S 1A1, Canada; eleanor.pullenayegum@sickkids.ca; 3 Canadian Centre for Applied Research in Cancer Control (ARCC), Toronto, ON M4N 3M5, Canada; 4 Child Health Evaluative Sciences, The Hospital for Sick Children, Toronto, ON M5G 1X8, Canada

**Keywords:** equity, EQ-5D-3L, health utility, oncology

## Abstract

Background: EQ-5D-3L health utilities inform health technology assessments in oncology, often derived from clinical trials enrolling highly selected populations, which may over-estimate real-world health utilities. Little is known about the association between socioeconomic status (SES) and EQ-5D-3L health utilities. Our objective was to examine EQ-5D-3L health utilities across SES in a real-world sample of patients with cancer. Methods: We conducted a cross-sectional analysis of EQ-5D-3L responses from 170 adult patients with cancer accrued from an Ontario oncology centre. We fitted multivariable linear regression models to estimate associations between covariates and EQ-5D-3L health utilities. Results: Lower EQ-5D-3L health utilities were significantly associated with the lowest and undisclosed family income categories in models with and without birth sex (*p* < 0.05). In the model including birth sex, disutility estimates for lowest family income (<CAD 29K) and undisclosed income was −0.202, 95% CI (−0.371 to −0.033), and −0.123 (−0.235 to −0.012), respectively. For the model excluding birth sex, disutility estimates for lowest income and undisclosed income was −0.163 (−0.280 to −0.046) and −0.106 (−0.184 to −0.028). Conclusions: Lower EQ-5D-3L health utilities were significantly associated with low income and nondisclosure, highlighting the need to incorporate SES in analyzing health utilities in cancer care.

## Simple Summary

Background and existing knowledge: Health utilities are a measure of health-related quality of life (HRQoL) used in cancer drug funding decisions. These are often derived from clinical trials with highly selected, socioeconomically advantaged participants, which can over-estimate HRQoL. To address this issue, we explored associations between EQ-5D-3L health utilities across a range of socioeconomic statuses in a real-world sample of patients with cancer. New findings: We found that HRQoL measured through EQ-5D-3L health utilities was lowest in patients in the lowest (under CAD 29,000) and undisclosed income categories. Implications: Our findings suggest that HRQoL measured through EQ-5D-3L health utilities may be associated with socioeconomic status, particularly family income. These findings can be used to support equity-informed health technology assessment.

## 1. Introduction

Health-related quality of life (HRQoL) is an important outcome in clinical decision-making and health economic evaluations in cancer care [1,2]. Health utilities are a quantitative measure of HRQoL, anchored at 0 (dead) and 1 (perfect health). Health utilities provide a weight on cancer-related survival to arrive at the quality-adjusted life year (QALY), used in cost–utility analysis, as part of health technology assessments (HTA) [3].

Health utilities used in HTA are often collected in pivotal clinical trials [4,5]. Participants enrolled in clinical trials are usually healthier [6] and are more socioeconomically advantaged than real-world patients [7,8]. Health utilities derived from these trials may therefore over-estimate the health utilities of those with lower socioeconomic status. There are important equity implications, since cost–utility analyses rely on trial-based health utilities, decisions arising from these analyses may be biased and favour interventions that were evaluated in these highly selected trial-based populations [9,10].

The EQ-5D questionnaire, developed by the EuroQol group (5), is the most common health utility instrument used in clinical trials and health technology assessments to measure HRQoL [11]. The EQ-5D-3L assesses five dimensions of HRQoL: mobility, self-care, usual activities, pain/discomfort, and anxiety/depression [12,13]. Each of these has three levels: no problems, some problems, or extreme problems/unable to perform activities [13]. The 5L version uses the same five dimensions, but has expanded to five levels: no problems, slight problems, moderate problems, severe problems, and unable to [14]. For both the EQ-5D-3L and EQ-5D-5L versions, an additional visual analogue scale (VAS) asks respondents to rate their current health from 0 (worst imaginable) to 100 (best imaginable) [15].

Socioeconomic status (SES), one’s access to material and social resources, aligns with the indicators used for socioeconomic position (SEP), in the World Health Organization’s social determinants of health conceptual framework [16]. These indicators (income, education, occupation), along with stratifiers such as social class, gender, and race/ethnicity, influence one’s exposure to advantage and disadvantage [16]. Social position determines health through intermediate factors, including material, psychosocial, behavioural, and biological factors [16], which impact HRQoL [17].

SES is associated with EQ-5D health utilities in oncology [18,19,20]. In Canada, a large survey of cancer survivors (*n* = 1759) found higher EQ-5D-3L health utilities among those with a university/college education compared with no university/college education *p* < 0.001, and lower health utilities among individuals not married compared with married or common law (*p* = 0.001). The same study found a large difference in mean (SE) health utilities by cancer site, from 0.76 (0.03) in pancreatic cancer to 0.90 (0.05) in chronic lymphocytic leukemia [18]. In China, among a sample of patients with colorectal cancer, lower household income was associated with lower EQ-5D-5L health utility, with a mean (SD) of 0.505 (0.419) for <¥20K to 0.759 (0.315) for >¥80K. These examples illustrate how factors such as marital status, income, education level, and cancer site have been associated with EQ-5D-3L or 5L responses in Canada and China [18,19].

In the Canadian cancer care space, health utilities are often collected in clinical trials under highly monitored conditions [21], yet there is no routine collection of real-world health utilities, which could improve patient care and facilitate real-world cost–utility analyses to inform health technology assessments.

Our team recently conducted a feasibility study of implementing the EQ-5D-3L questionnaire at a pilot oncology centre in Ontario, Canada [22], enabling us to examine how patient characteristics and SES impact EQ-5D-3L health utilities. We used the EQ-5D-3L instrument instead of the more recent EQ-5D-5L version, as the 3L version has more historic use than the 5L version as a clinical outcome assessment in health technology assessment, regulatory reviews, and systematic literature reviews [23]. The objective of this current study was to examine patient characteristics that may be responsible for differences in EQ-5D-3L health utilities in a real-world sample of patients with cancer. We hypothesized that patient characteristics of age, sex, education, marital status, employment status, income, primary cancer site, and ethnicity would be associated with EQ-5D-3L health utilities.

## 2. Materials and Methods

### 2.1. Study Design and Population

This was a cross-sectional analysis of initial EQ-5D-3L responses from patients with cancer. We accrued a prospective convenience sample of patients with any solid tumour or hematological malignancy to complete the EQ-5D-3L during their chemotherapy appointment at the Sunnybrook Odette Cancer Centre in Toronto, Ontario, Canada. Eligible patients were 18 years or over, starting any publicly reimbursed systemic therapy, and provided informed consent. Along with completing the EQ-5D-3L and EQ-VAS, patients were invited to complete a demographic questionnaire. The patient’s primary cancer site was abstracted from their visit record at screening. Patients were accrued from May to November 2024, with patient accrual described in greater detail in our earlier manuscript [20].

### 2.2. Study Sources and Measures

The EQ-5D-3L health utility score was the outcome variable of interest in this study. We included covariates comprising patient demographics and clinical characteristics identified from the literature [18,24], conceptual relevance, and refined based on expert opinion (KC). The specific variables tested in our models were age, sex, education, marital status, employment status, family income, ethnicity, and primary cancer site. Age was tested as both a continuous and categorical variable in separate models. Models with and without the birth sex variable were tested to assess the effect of sex-specific cancers. First, we included data with all cancers and excluded sex as a variable (*n* = 170). To incorporate sex as a variable, we excluded individuals with gynecological, breast, and prostate cancers in our multivariable analysis (*n* = 111). These sample sizes allowed the estimation of model parameters and their 95% confidence intervals (CIs) with reasonable precision, according to linear regression best practices [25], and the accuracy in parameter estimation principle [26].

### 2.3. Statistical Analysis

We conducted ordinary least squares (OLS) multivariable regression to estimate the association between multiple covariates and EQ-5D-3L utility scores.

We tested main effects models followed by models with interactions between birth sex and age categories (<50, 50 to 74, >75 years) based on conceptual relevance. Main effects models and models with interactions were assessed using analysis of variance (ANOVA) to evaluate the significance of the interaction term. Nested models were compared using the Bayesian Information Criterion (BIC), where a smaller BIC represents a simpler model. Reference categories were assigned based on either the largest sample size or the group thought to be most privileged, based on the literature [27,28]. Because the calculated health utilities were left-skewed (Supplementary material Figure S1) and income was categorizeed into ordinal levels, Spearman’s rank correlations were explored between numerical income categories (<CAD 29,999 to >CAD 150,000) and each EQ-5D-3L dimension. Positive coefficients indicate that lower income is associated with higher problem severity.

### 2.4. Software

All analyses were conducted using R version 4.5.0. EQ-5D-3L health utilities were scored using the eq5d package (version 0.15.7), with the Canadian EQ-5D-3L time-trade-off (TTO) scoring model [29]. The best and worst health states for this Canadian TTO value set ranged from 1.000 for 11,111 (best health) to a predicted mean (SE) −0.340 (0.013) for 33,333 (worst health) [30].

## 3. Results

### 3.1. Participant Characteristics

A summary of patient characteristics is presented in Table 1. Most respondents identified as White—108 (65.5%)—and a large percentage completed at least some college or university education—127 (75.6%). Most respondents were married or in a common-law relationship—115 (68.9%)—and half were retired—83 (50.3%). Of patients who reported a family income, 29 (17.3%) reported an annual family income of over CAD 150,000; however, 79 (46.5%) did not report a family income. In the full sample, amongst those with a reported primary cancer site, the gynecological cancers were most common—40 (23.5%)—whereas head and neck cancers were the most common amongst cancers affecting all sexes—32 (18.8%). As the second most frequent cancer, head and neck cancers were assigned as the reference category for primary cancer sites, to ensure that models with all cancers and models without sex-specific cancers could be compared.

### 3.2. EQ-5D-3L Health Utility by Cancer Site

Table 2 shows descriptive statistics of mean health utility (SD) for the different cancer sites. The primary cancer sites with the highest mean health utilities (SD) were colorectal—0.918 (0.127); skin—0.819 (0.096); and breast—0.815 (0.162). In contrast, the primary cancer sites with the lowest mean health utilities (SD) were upper gastrointestinal—0.731 (0.127); genitourinary—0.717 (0.174); and thoracic—0.712 (0.254).

### 3.3. Association Between EQ-5D-3L Dimension and Income

**Figure 1:**
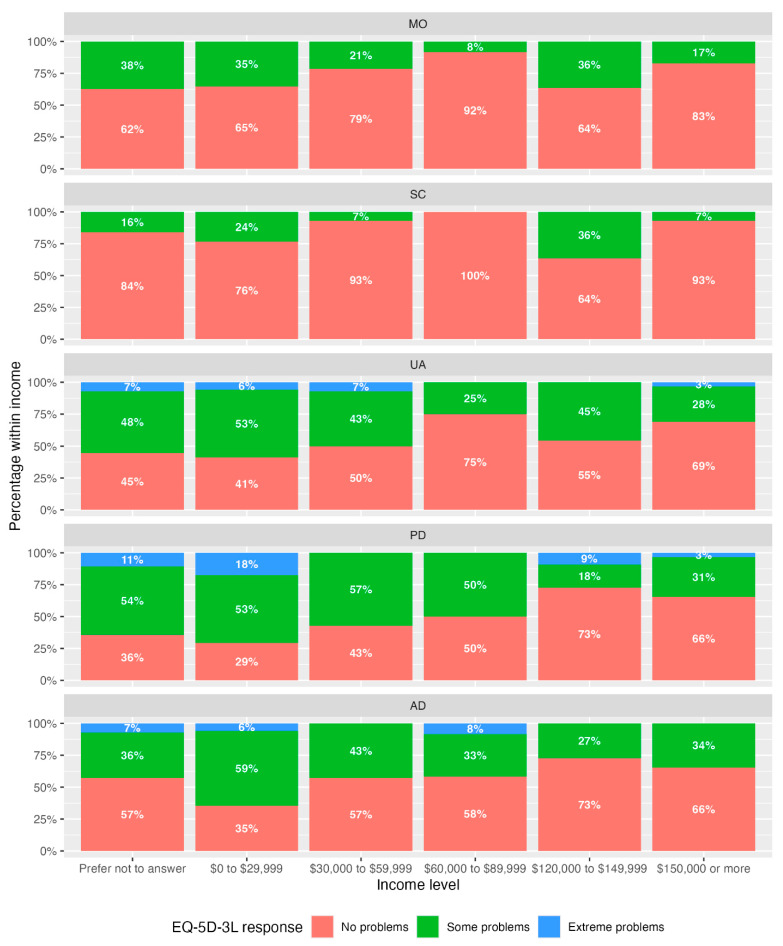
Stacked bar plots of percentages of response level by dimension and income category. No participants selected income category CAD 90,000 to 119,999.

Table 3 shows the Spearman’s rank correlation (ρ) between EQ-5D-3L dimensions and income. Figure 1 is a stacked bar plot of income level and the percentage of patients within each income level. The dimensions pain/discomfort (PD) and anxiety/depression (AD) were significantly associated with lower income (ρ PD = 0.291, *p* = 0.008; ρAD = 0.219, *p* = 0.046).

Stacked bar plots (Figure 1) illustrate a higher percentage of responses of “some problems” and “extreme problems” in the income groups “prefer not to answer” and CAD 0 to 29,999, particularly for dimensions UA, PD, and AD. The largest percentage of “extreme problems” was seen within the “prefer not to answer” (11%) income category and CAD 0 to 29,999 (18%) category, both for the PD dimension. These percentages are in agreement with Spearman’s correlation.

### 3.4. Patient Demographics Associated with Health Utility

Table 4 presents the results of our multivariable regression models of patient demographic factors associated with EQ-5D-3L health utility. The two models presented are (i) one including birth sex and excluding participants with no sex-specific cancers (*n* = 111) and (ii) one excluding birth sex and including all cancers (*n* = 170). Model fit statistics of all fitted models, including those with interactions are presented in the Supplementary materials Table S1. 

In both the models with and without birth sex, a family income of CAD 0 to 29K and undisclosed income were associated with a significantly lower health utility (*p* < 0.05). The model with birth sex had a disutility estimate for income < CAD 0 to 29K (95% CI) of −0.202 (−0.371 to −0.033), and undisclosed income had a disutility estimate of −0.123 (−0.235 to −0.012). The model that excludes birth sex had a disutility estimate for income CAD 0 to 29K (95% CI) of −0.163 (−0.280 to −0.046), and undisclosed income had a disutility estimate of −0.106 (−0.184 to −0.028).

Of all primary cancer sites, those with colorectal cancers had significantly higher utility estimates (*p* < 0.05) by 0.135 (0.010 to 0.260) for the model with the sex variable, and 0.147 (0.031 to 0.263) for the model excluding the sex variable.

The interaction term between age as a categorical variable and birth sex was not statistically significant. Models with interaction terms between birth sex and age found that tests of significance of the interaction terms were not significant (*p* = 0.359) (See Supplementary materials Tables S2 and S3).

## 4. Discussion

This study found significant associations between income and EQ-5D-3L health utility. Individuals in the lowest family income category (CAD 0 to 29,999) and those who did not disclose their income had significantly lower health utilities than those with a family income of at least CAD 150,000. Our sample of participants included participants with birth sex distributions similar to those with cancer in Canada [32], participants of a higher average age than in Ontario (our study: 64.5; Ontario: 41.3 years) [33], and more participants with a family income > CAD 150,000 compared with Ontario (our study: 17.1%; Ontario: 15.4%) [34]. Participants in the lowest family income category in our study were within the lowest 10% of after-tax income for people in an economic family or the lowest 20% of people not in an economic family in Ontario, Canada [35]. The resulting health utility gaps between those in the lowest and highest income categories are relevant to HTA decisions.

Income has been associated with EQ-5D-3L health utilities across a number of countries. Janssen et al. reported population norms of EQ-5D-3L in 20 countries, finding that macroeconomic factors including prior living standards, represented by GDP per capita and health expenditure per capita, were positively correlated with mean EQ-VAS scores at 0.58 and 0.55, respectively [27]. In a population-level study from Brazil, participants in intermediate and lower SES classes had significantly lower EQ-5D-3L health utilities compared with the higher SES classes, regardless of their age, sex, and education, based on a multivariable analysis [20]. These findings are in agreement with our findings that being in the lowest income category is associated with lower health utility.

In Canada, like other countries with a universal health care system, the societal costs of cancer include both health care system costs as well as costs borne by people with cancer and their caregivers [36]. People diagnosed with cancer, along with their caregivers, experience direct costs, indirect costs, and psychosocial costs, where the latter includes health-related quality of life costs [36]. Population norms for EQ-5D-3L have consistently found that individuals in a low-income category have lower health utilities, independent of their health status [20,27,37,38]. We hypothesize that individuals in a lower income category may experience further decrements to their health utility as a consequence of the financial impacts of a cancer diagnosis. This is consistent with our findings of positive associations between low income and high problem-severity level, particularly in the pain/discomfort and anxiety/depression dimensions.

Our findings add to the larger body of literature on the association of family income with health utilities amongst people with cancer. The findings from this study are comparable with another large study on cancer health utilities collected in Ontario, Canada [18]. Naik et al. analyzed EQ-5D-3L responses from 1759 ambulatory patients with cancer, calculated EQ-5D-3L scores across 26 cancers, and constructed a multivariable model to establish an association of factors influencing EQ-5D-3L response. A notable difference was that our sample of patients with colorectal cancers had higher EQ-5D-3L health utilities (SD)—0.918 (0.127)—compared with Naik—0.83 (0.107) [18]. Patients presenting with colorectal cancer in our study had the highest EQ-5D-3L health utilities, potentially because of long-term survivorship or positive response to treatment. Of the cancer sites that were similarly categorized, mean health utilities were comparable between our studies [18]. A key methodological difference is that Naik et al. did not include age, sex, and income as variables in their multivariable model. They reported that age was not a significant variable (*p* = 0.54) and excluded sex as a variable because of the inclusion of sex-specific cancers. In contrast, our study incorporated SES variables including age, sex, and income to explore equity-related response heterogeneity in the EQ-5D-3L, which has not been previously studied in the Canadian cancer care context.

There are strengths and limitations to our study. A major strength of our findings is our contribution to the association of patient characteristics with cancer health utilities. We found an association between individuals in a lower income category or who prefer not to report their income and lower health utilities. Our sample collected birth sex and gender, and noted complete concordance between the two, largely owing to our small sample size. Broader implementation of the EQ-5D across Ontario would allow any gender specific analyses to be properly completed. Another strength of our findings is in adding to the knowledge on EQ-5D-3L health utilities in the Canadian context to provide input parameters for future cost–utility analyses. There are several limitations of our study. First, using EQ-5D-3L to measure health utilities is susceptible to ceiling effects [39], which can under-estimate overall population-level health utilities. These ceiling effects are thought to be reduced by using the EQ-5D-5L, which also has improved responsiveness [39]. In a sample of patients with cancer from Iran, the EQ-5D-5L demonstrated lower ceiling effects compared with the EQ-5D-3L. Ceiling effects were observed for both EQ-5D-3L and EQ-5D-5L (12.07% and 9.44%, respectively), both lower than the acceptable limit of 15% for health status questionnaires [40]. We did not account for unmeasured confounding in this study, including comorbidities or cancer stage, which have been shown to influence EQ-5D-3L health utilities [18]. Our model that includes the birth sex variable (28 df, *n* = 111) has wider 95% CI estimates, even though it satisfies model parsimony and clinical relevance. Lastly, we conducted a cross-sectional pilot single-site study in an urban Canadian oncology centre, which had a small sample size (*n* = 170 full sample; *n* = 111 sample excluding sex-specific cancers). The impact of our smaller sample size is that all health utility estimates are susceptible to wider standard errors, reflecting the uncertainty in measurement.

There are implications of our work for users of EQ-5D-3L health utilities. Our findings suggest that HTAs that use trial-based health utilities may be over-estimated, yet the implications on incremental QALYs gained from novel therapies are unclear. We therefore encourage health economists, researchers, and policy makers to consider the association of patient characteristics and SES, specifically income, with health utilities used in economic models for HTA. Incorporating real-world health utilities, which account for equity-related factors, can more judiciously allocate scarce health care resources, so that health care decisions can better reflect the HRQoL of equity-deserving populations. Future research can explore longitudinal health utility collection to understand the association of equity-deserving variables on change in health utilities from initial diagnosis onwards, and their applications on distributional cost-effectiveness analyses [41].

## 5. Conclusions

This study provides the first Canadian real-world estimates that quantify the effect of income on EQ-5D-3L health utilities in oncology. Patient demographic characteristics and SES, in particular low income, are associated with lower EQ-5D-3L health utilities.

## Figures and Tables

**Table 1:** Baseline characteristics of study participants.

Variable	Study Participants (*n* = 170); Number (%)
Sex	
Male	71 (41.8%)
Female	96 (56.5%)
Age	
Mean (standard deviation)	64.5 (12.9)
Range	23 to 99
<50	23 (13.5%)
50 to 74	111 (65.3%)
75 to 99	32 (18.8%)
Not disclosed	4 (2.4%)
Education	
Did not attend college or university	38 (22.4%)
Attended college or university	127 (74.7%)
Other	3 (1.8%)
Not disclosed	2 (1.2%)
Marital status	
Married or common law	115 (67.6%)
Other	55 (32.4%)
Employment status	
Working full-time	43 (25.3%)
Other	25 (14.7%)
Unemployed	7 (4.1%)
Working part-time	10 (5.9%)
Retired	83 (48.8%)
Not disclosed	2 (1.2%)
Family Income *	
<CAD 29,999	17 (10.0%)
CAD 30,000–59,999	14 (8.2%)
CAD 60,000–89,999	12 (7.1%)
CAD 90,000–119,999	0 (0.0%)
CAD 120,000–149,999	11 (6.5%)
>CAD 150,000	29 (17.1%)
Do not know	21 (12.4%)
Prefer not to answer	56 (32.9%)
Missing	10 (5.9%)
Ethnicity	
White	108 (63.5%)
East, SE, or South Asian **	48 (28.2%)
Black	4 (2.4%)
Other population/race ***	7 (4.1%)
Prefer not to answer	3 (1.8%)
Primary Cancer Site	
Breast	18 (10.6%)
Colorectal	12 (7.1%)
Genitourinary	5 (2.9%)
Gynecological	40 (23.5%)
Head and Neck	32 (18.8%)
Hematological	17 (10.0%)
Skin	9 (5.3%)
Thoracic	12 (7.1%)
Upper Gastrointestinal	13 (7.6%)
Other ****	12 (7.1%)

* Annual household income refers to fiscal year 2024 in Canadian dollars (CAD). ** All Asians were combined into one group, comprising East Asian, South Asian, Southeast Asian. *** Ethnicities in the other population/race group comprising responses from individuals who selected Other, Indigenous, Latin American, and Middle Eastern as per CIHI’s Guidance on the Use of Standards for Race-Based and Indigenous Identity Data Collection and Health Reporting in Canada [31]. **** Other primary cancer sites included cancers of the central nervous system, neuroendocrine cancers, other cancers, and missing cancer sites.

**Table 2:** Cancer site, mean health utility (SD), and number of patients.

Primary Cancer Site	n	Mean Utility	SD Utility
Colorectal	12	0.918	0.127
Skin	9	0.819	0.096
Breast	18	0.815	0.162
Hematological	17	0.802	0.172
Head and Neck	32	0.757	0.170
Gynecological	40	0.752	0.164
Upper Gastrointestinal	13	0.731	0.127
Genitourinary	5	0.717	0.174
Thoracic	12	0.712	0.254
Other *	12	0.848	0.141

* Other categorization includes central nervous system, neuroendocrine, other, and missing cancer sites.

**Table 3:** EQ-5D-3L dimension and Spearman’s rank correlation with income level.

Dimension	Spearman’s ρ	*p*-Value
MO	0.114	0.306
SC	0.103	0.353
UA	0.199	0.071
PD	0.291	0.008
AD	0.219	0.046

MO: mobility. SC: self-care. UA: usual activity. PD: pain and discomfort. AD: anxiety and depression.

**Table 4:** EQ-5D-3L health utility as the outcome predicted by socioeconomic status: two main effects models with and without sex-specific cancers.

	Model Including Birth Sex Variable, No Participants with Sex-Specific Cancers (*n* = 111)			Model Excluding Birth Sex Variable, Including Participants with All Cancers (*n* = 170)		
Variable	Estimate	95% CI	*p*-Value	Estimate	95% CI	*p*-Value
(Intercept)	0.866	(0.729 to 1.002)	<0.001 ***	0.811	(0.718 to 0.903)	<0.001 ***
Age						
<50	−0.029	(−0.156 to 0.099)	0.655	−0.035	(−0.118 to 0.049)	0.411
50 to 74	Reference					
75 to 99	0.047	(−0.046 to 0.141)	0.317	0.005	(−0.070 to 0.080)	0.899
Sex						
Female	Reference					
Male	−0.032	(−0.105 to 0.040)	0.377	NA		
Education						
Did not attend college/ university	0.003	(−0.088 to 0.094)	0.953	0.016	(−0.053 to 0.084)	0.652
Attended college or university	Reference					
Other	0.060	(−0.193 to 0.313)	0.637	0.057	(−0.142 to 0.256)	0.571
Marital status						
Married or common law	Reference					
Other	0.032	(−0.058 to 0.122)	0.480	0.036	(−0.026 to 0.098)	0.258
Employment status						
Working part-time	−0.018	(−0.187 to 0.152)	0.836	0.060	(−0.067 to 0.187)	0.353
Working full-time	Reference					
Other	−0.043	(−0.172 to 0.087)	0.515	−0.042	(−0.127 to 0.043)	0.326
Unemployed	0.039	(−0.151 to 0.230)	0.682	0.062	(−0.076 to 0.200)	0.376
Retired	−0.013	(−0.114 to 0.089)	0.804	0.017	(−0.053 to 0.088)	0.628
Income						
CAD 0–29K	−0.202	(−0.371 to −0.033)	0.020 *	−0.163	(−0.280 to −0.046)	0.007 **
CAD 30K–59K	−0.049	(−0.193 to 0.095)	0.503	−0.043	(−0.160 to 0.075)	0.474
CAD 60K–89K	0.014	(−0.142 to 0.169)	0.859	−0.013	(−0.131 to 0.104)	0.822
CAD 120K–149K	−0.032	(−0.177 to 0.113)	0.664	−0.041	(−0.160 to 0.078)	0.496
>CAD 150K	Reference					
Do not know	−0.053	(−0.188 to 0.081)	0.433	−0.046	(−0.149 to 0.057)	0.377
Prefer not to answer	−0.123	(−0.235 to −0.012)	0.031 *	−0.106	(−0.184 to −0.028)	0.008 **
Primary cancer site						
Head and neck	Reference					
Gynecological	NA			0.018	(−0.064 to 0.099)	0.670
Breast	NA			0.070	(−0.028 to 0.169)	0.162
Colorectal	0.135	(0.010 to 0.260)	0.034 *	0.147	(0.031 to 0.263)	0.013 *
Genitourinary ^@^	−0.118	(−0.315 to 0.079)	0.237	−0.066	(−0.230 to 0.097)	0.425
Hematological	0.043	(−0.078 to 0.163)	0.483	0.051	(−0.055 to 0.156)	0.346
Other	0.022	(−0.114 to 0.157)	0.753	0.035	(−0.092 to 0.162)	0.584
Skin	0.018	(−0.125 to 0.162)	0.801	0.043	(−0.090 to 0.175)	0.526
Thoracic	−0.033	(−0.159 to 0.094)	0.609	−0.024	(−0.139 to 0.091)	0.678
Upper gastrointestinal	−0.017	(−0.141 to 0.107)	0.785	−0.006	(−0.118 to 0.106)	0.912
Ethnicity						
White	Reference					
East/SE/South Asian	−0.015	(−0.097 to 0.068)	0.727	−0.017	(−0.076 to 0.043)	0.577
Other/Not Identified Elsewhere (NIE)	−0.046	(−0.256 to 0.163)	0.660	−0.089	(−0.229 to 0.051)	0.213
Black ^#^	−0.375	(−0.732 to −0.017)	0.040 *	−0.066	(−0.236 to 0.104)	0.444

Significance levels: * *p* < 0.05, ** *p* < 0.01, *** *p* < 0.001. ^#^ One participant identified with being of black ethnicity; therefore, the estimate is very uncertain. ^@^ One participant with prostate cancer was removed from the analysis with *n* = 111 patients.

## Data Availability

The raw data supporting the conclusions of this article can be made available by the authors on request.

## Supplementary Materials

The following supporting information can be downloaded at https://www.mdpi.com/article/10.3390/curroncol32110645/s1. Figure S1. Histogram distribution of EQ-5D-3L health utility scores. Table S1. Table of multivariable model fit statistics. Table S2. Table of age (categorical)/birth sex interaction, no participants with female and male cancers. Table S3. Table of birth sex/age (categorical) interaction, no participants with female and male cancers.

## Abbreviations

The following abbreviations are used in this manuscript:

AD	Anxiety and depression
ANOVA	Analysis of variance
BIC	Bayesian Information Criterion
CI	Confidence interval
HRQoL	Health-related quality of life
HTA	Health technology assessment
MO	Mobility
OLS	Ordinary least squares
PD	Pain and discomfort
QALY	Quality-adjusted life year
SC	Self care
SD	Standard deviation
SES	Socioeconomic status
TTO	Time trade off
UA	Usual activity
VAS	Visual analogue scale
