# Supplementary material for: Examining the Association Between Equity-Related Factors and EQ-5D-3L Health Utilities of Patients with Cancer
Source: Curr Oncol. 2025 Nov 19;32(11):645. doi: 10.3390/curroncol32110645 (PMC12651012; doi:10.3390/curroncol32110645)
Supplement: Supplementary file 1 [file curroncol-32-00645-s001.zip › curroncol-3931559-supplementary.pdf]

Supplementary Materials

Figure S1. Distribution of EQ-5D-3L health utility scores

Table S1. Table of multivariable model fit statistics

Table S2. Table of age (categorical) / birth sex interaction, no participants with female and male cancers

Table S3. Table of birth sex / age (categorical) interaction, no participants with female and male cancers

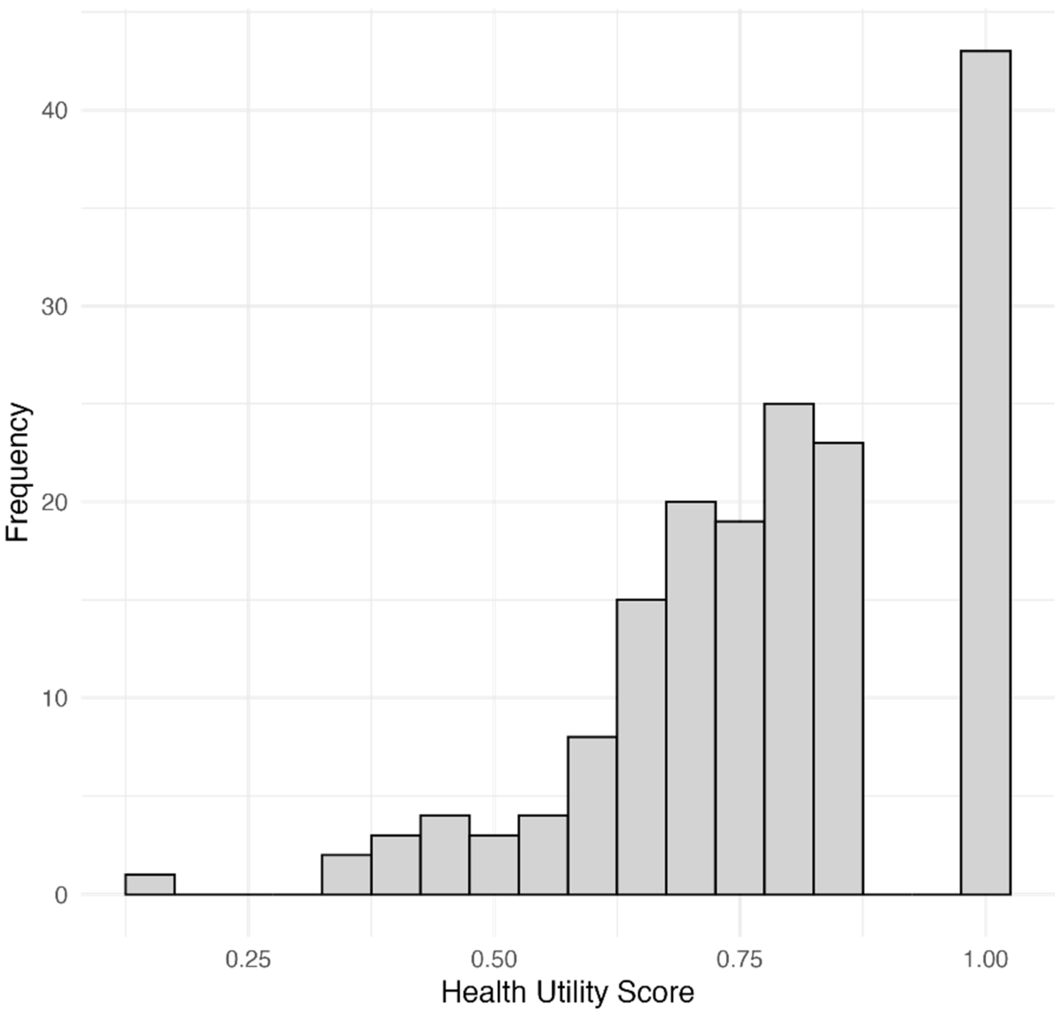

Figure S1. Histogram distribution of EQ-5D-3L scores.

Table S1. Table of multivariable model fit statistics.

Model	Unique characteristic	AIC	BIC
	Main effects models		
m6_v4_no_interact (Table 4 in main text)	Age (categorical), birth sex, no participants with female and male cancers (n = 111)	-59.73	15.63
m6_v5 (Table 4 in main text)	Age (categorical), no birth sex variable, all cancers (n = 170)	-103.34	-14.15
	Additional models with interactions		
m6_v4	Age (categorical) / birth sex interaction, no participants with female and male cancers (n = 111)	-58.52	22.22
m6_v6	Birth sex / age (categorical) interaction, no participants with female and male cancers (n = 111)	-58.52	22.22

Table S2. Table of model with age (categorical) / birth sex interaction, no participants with female and male cancers (n = 111).

Variable	Estimate	2.5% CI	97.5% CI	p-value	Sig
(Intercept)	0.857	0.714	1.000	<0.001	***
Age					
< 50	-0.089	-0.289	0.112	0.381	
50 to 74	Reference				
75 to 99	0.093	-0.055	0.242	0.216	
Education					
Did not attend college/university	-0.008	-0.1	0.084	0.863	
Attended college of university	Reference				
Other	0.079	-0.178	0.336	0.543	
Marital status					
Married or common law	Reference				
Other	0.028	-0.064	0.12	0.547	
Employment status					
Working full time	Reference				
Other	-0.035	-0.166	0.096	0.597	
Unemployed	0.036	-0.155	0.227	0.708	
Working part-time	-0.004	-0.177	0.168	0.962	
Retired	-0.006	-0.108	0.096	0.905	
Family income					
$0–$29K	-0.18	-0.352	-0.008	0.04	*
$30K–$59K	-0.039	-0.186	0.107	0.596	
$60K–$89K	0.013	-0.142	0.169	0.867	
$120K–$149K	-0.03	-0.175	0.115	0.678	
>$150K	Reference				
Do not know	-0.043	-0.179	0.093	0.529	
Prefer not to answer	-0.126	-0.238	-0.014	0.028	*
Primary cancer site					
Head and neck	Reference				
Colorectal	0.142	0.017	0.267	0.027	*
Genitourinary	-0.095	-0.296	0.106	0.348	
Hematological	0.035	-0.086	0.155	0.572	
Other	0.016	-0.12	0.152	0.819	
Skin	0.019	-0.124	0.163	0.792	
Thoracic	-0.028	-0.155	0.098	0.658	
Upper GI	-0.006	-0.137	0.125	0.926	
Ethnicity					
White	Reference				
East/SE/South Asian	-0.021	-0.104	0.063	0.623	
Other/Not Identified Elsewhere (NIE)	-0.063	-0.273	0.148	0.556	
Black	-0.355	-0.717	0.006	0.054	.
*Age x birth sex interaction^*					
Age < 50 × Male	0.076	-0.123	0.275	0.447	
Age 50–74 × Male	-0.028	-0.123	0.067	0.56	
Age 75–99 × Male	-0.099	-0.248	0.05	0.19	

Significance levels: *p <0.05, **p<0.01, ***p<0.001; ^For each age x birth sex interaction term, the reference is females of the same age range.

Table S3. Table of model with birth sex / age (categorical) interaction, no participants with female and male cancers (n = 111).

Predictor	Estimate	2.5% CI	97.5% CI	p-value	Sig
(Intercept)	0.857	0.714	1	<0.001	***
Sex					
Female	Reference				
Male	-0.028	-0.123	0.067	0.56	
Education					
Did not attend college/university	-0.008	-0.1	0.084	0.863	
Attended college or university	Reference				
Other	0.079	-0.178	0.336	0.543	
Marital status					
Married or common law	Reference				
Other	0.028	-0.064	0.12	0.547	
Employment status					
Working full time	Reference				
Other	-0.035	-0.166	0.096	0.597	
Unemployed	0.036	-0.155	0.227	0.708	
Working part-time	-0.004	-0.177	0.168	0.962	
Retired	-0.006	-0.108	0.096	0.905	
Family income					
$0–$29K	-0.18	-0.352	-0.008	0.04	*
$30K–$59K	-0.039	-0.186	0.107	0.596	
$60K–$89K	0.013	-0.142	0.169	0.867	
$120K–$149K	-0.03	-0.175	0.115	0.678	
>$150K	Reference				
Do not know	-0.043	-0.179	0.093	0.529	
Prefer not to answer	-0.126	-0.238	-0.014	0.028	*
Primary cancer site					
Head and neck	Reference				
Colorectal	0.142	0.017	0.267	0.027	*
Genitourinary	-0.095	-0.296	0.106	0.348	
Hematological	0.035	-0.086	0.155	0.572	
Other	0.016	-0.12	0.152	0.819	
Skin	0.019	-0.124	0.163	0.792	
Thoracic	-0.028	-0.155	0.098	0.658	
Upper GI	-0.006	-0.137	0.125	0.926	
Ethnicity					
White	Reference				
East/SE/South Asian	-0.021	-0.104	0.063	0.623	
Other/Not Identified Elsewhere (NIE)	-0.063	-0.273	0.148	0.556	
Black	-0.355	-0.717	0.006	0.054	.
*Birth sex x Age interaction^*					
Female × Age < 50	-0.089	-0.289	0.112	0.381	
Male × Age < 50	0.016	-0.131	0.162	0.831	
Female × Age 75 to 99	0.093	-0.055	0.242	0.216	
Male × Age 75 to 99	0.022	-0.093	0.137	0.703	

Significance levels: *p <0.05, **p<0.01, ***p<0.001; ^For each birth sex x age interaction term, the reference is people of the corresponding birth sex who are 50 to 74 years.
